# Effective prediction finite element model of pull-out capacity for cast-in-place anchor in high strain rate effects

**DOI:** 10.1038/s41598-023-44510-y

**Published:** 2023-10-23

**Authors:** Quoc To Bao, Kihak Lee, Hyoseo An, Do Hyung Lee, Jiuk Shin

**Affiliations:** 1 Department of Architectural Engineering, Deep Learning Architecture Research Center, Sejong University, 209 Neungdong-ro, Gwangjin-gu, Seoul, 05006 Republic of Korea; 2 Department of Civil, Environmental and Railroad Engineering, PaiChai University, Daejeon, 302-735 South Korea; 3 Department of Architectural Engineering, Gyeongsang National University, Jinju, 52828 Republic of Korea

**Keywords:** Civil engineering, Mechanical engineering

## Abstract

Cast-in-place anchors are being increasingly used in many applications including building construction, bridge, and power plants. The anchorage to concrete systems are subjected to tensile, shear and combined loads from a variety of loading circumstances including static, dynamic, and shock loading. Despite extensive studies on these systems, reliable numerical models for predicting the behavior of these anchors are still limited. Therefore, this paper investigated the tensile behavior of cast-in-place anchorage to concrete systems, to propose an effective model for reproducing anchorage behavior using finite element (FE) methods. Experiments and code-based models for the anchorage system in tension were used to evaluate the numerical models for cast-in-place anchors in concrete, and the most suitable model, with advantages in accuracy and saving analysis time, was chosen. Finally, the FE model was used to study the tensile capacity and related dynamic increase factor for various strain rates, anchor diameters, and embedment depths.

## Introduction

Cast-in-place anchors are widely used in the building of nuclear power plants, buildings, and bridges. These anchorage systems are subjected to shear, tensile, and combination loads that may be static or dynamic, including seismic, impact, and blast loading. Numerous studies have examined the potential effects of factors on the tensile or shear behavior of the anchors, such as edge distance, anchor diameter, embedment depth, and anchor spacing. One of the most crucial factors is embedment depth since it impacts the pull-out strength and the projected locations where the anchors break in a cone shape when subjected to tensile load^1–3^.

In particular in the anchorage zone, pull-out tests are required to understand how anchors and concrete interact. In order to as precisely as possible mimic designed or as-built conditions, the test technique is meant to be a performance test. Research on the pull-out strength of anchors under static and dynamic tensile loading has been published^4^. The pull-out strength is significantly affected by anchor embedment depths, as well as anchor diameter. Concrete cone breakout failure at embedment depths of 80 mm, 100 mm, 120 mm, and 130 mm and failure behavior for steel at 310 mm embedment depth were also evaluated^5,6^. The pull-out strength is an important parameter to the bond-slip effect and is significant for predicting the bond-slip behavior of the entire structure. The equations for predicting bond strength under various conditions (e.g., embedment length, concrete strength, bar diameter, etc.) have been proposed in previous research^7–13^.

**Figure 1:**
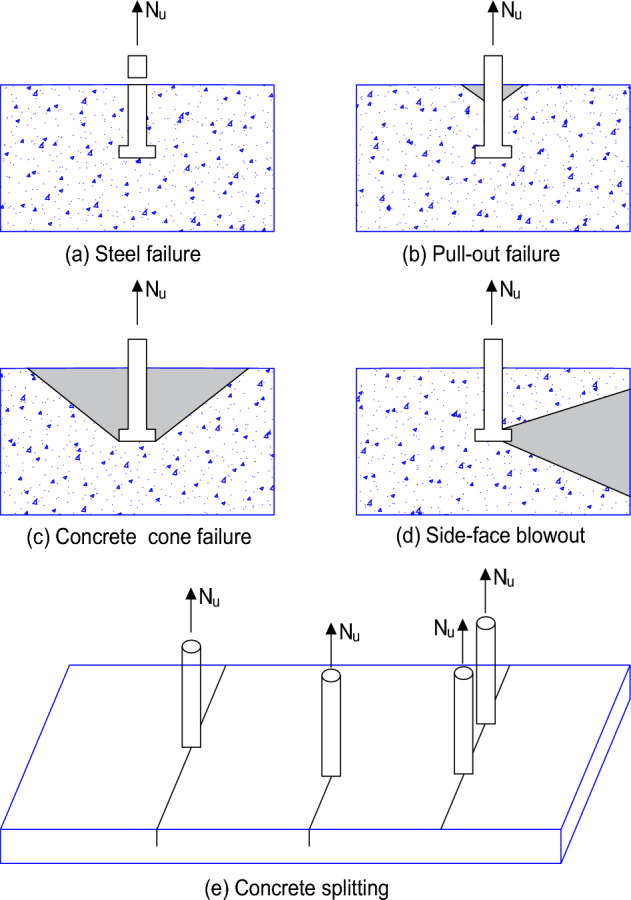
Failure models of single cast-in-place anchors under tensile loading.

When a steel anchor is subjected to tensile loading, five failure modes are typically observed: anchor bolt failure, concrete cone breakout failure, side face blowout failure, concrete splitting, and anchor pull-out. Anchor bolt failure happens when the strength of the anchor is less than the applied tensile stress or the anchor strength is lower than the ultimate load for the other models of failure. Concrete cone breakout failure occurs at shallow embedment depths when the tensile stress applied to the anchor bolt exceeds the strength of the concrete. When there is not enough edge spacing, side face blowout failure occurs. Due to insufficient concrete member depth, concrete splitting occurs when the tensile strength of the concrete is less than the bolt strength. When the friction between the anchor and the concrete is less than the applied tensile load for the anchors without head, an anchor pullout failure occurs^14,15^. Figure 1 shows the failure modes for the cast-in-place anchors under tensile loading.

Numerous previous experimental investigations and simulation results have examined the behavior of deformed bars removed from a concrete block under monotonic loading. Most published research has found the ascending branch of the bond-slip curve to be the most important part, and prediction programs that have analytically described pull-out behavior have only been based on the mechanical contact component^16–22^.

In order to minimize the quantity of experimentation, a numerical simulation is used with experimental studies to better understand the influence of various features and parameters. Due to the considerable development of these models and software applications over the last few decades, the use of numerical techniques to predict bond-slip interface behavior based on the FE method has dramatically expanded. A perfect bond, which prevents any relative slip between the reinforcing bar and concrete, was taken into account by several authors when modeling the interface^23–28^. Recently, non-perfect bond models that pay particular attention to the mechanics of mechanical interaction and friction have been reported^29–32^.

There has been limited investigation of the effects of dynamic loads including impacts and blast on the anchorage of concrete systems, compared to the studies that have examined the effects of dynamic loads on concrete or steel materials. Tedesco et al.^33^ investigated FE research on concrete under compressive load. Based on the previous studies, strain rate had significant influence on the kind of failure behavior of concrete at a 17 $\mathrm {~s}^{-1}$ of strain rate. The failure of concrete was observed around 35% for the concrete specimen at a strain rate of 25 $\mathrm {~s}^{-1}$, which was when cracking began to occur. Moreover, at 200 $\mathrm {~s}^{-1}$ of strain rate, there was about 85% concrete failure observed. Additionally, the tensile strength of concrete is more sensitive to strain rate than is the compressive strength, according to Min et al.^34^. Based on the previous studies, the dynamic increase factor (DIF), which is a bilinear function of the strain rate, is critical for the development of structures under high strain rate.

Yu et al.^35^ and Lee et al.^36^ researched how strain rate affected the behavior of steel. Yu et al.^35^ investigated the influence of strain rate on DP600 steel (It is a cold-rolled, uncoated dual phase (DP) steel with a strength of approximately 6000 MPa) with strain rate from $10^{-4} \mathrm {~s}^{-1}$ to $10^{3} \mathrm {~s}^{-1}$. Because the DP600 steel was made of ferrite and martensite, they had a tensile strength greater than 600 MPa. The authors determined that the strain rate had significant effects on the steel’s mechanical behavior. Therefore, a novel constitutive model was presented by the authors to evaluate the mechanical behavior of steel. Additionally, the fracture reaction of stainless steel subjected to high strain rate was studied by Lee et al.^36^. The authors determined that the specimen’s composition and the stress-strain behavior are both effected by the strain rate. The degree of grain deformation increased together with the strain rate. According to the authors, the steel fractures were created by plastic instability at the adiabatic shear zones. This suggests that the loading speed on the anchorage of a concrete system can be the main variable determining bond-slip behavior.

The contact-based anchorage model or contact function is a general definition that can be used in most FE method programs. The one-dimensional (1D) slide line model, which can simulate bond-slip behavior similar to the real behavior, has been developed in the LS-DYNA program. As a result, the contact-based anchorage model has been widely used to examine earlier hypotheses concerning the interaction between cast-in place anchorage of concrete systems, and the 1D-slide line model has had certain limitations when it comes to simulating the behavior of cast-in-place anchorages of concrete systems.

**Figure 2:**
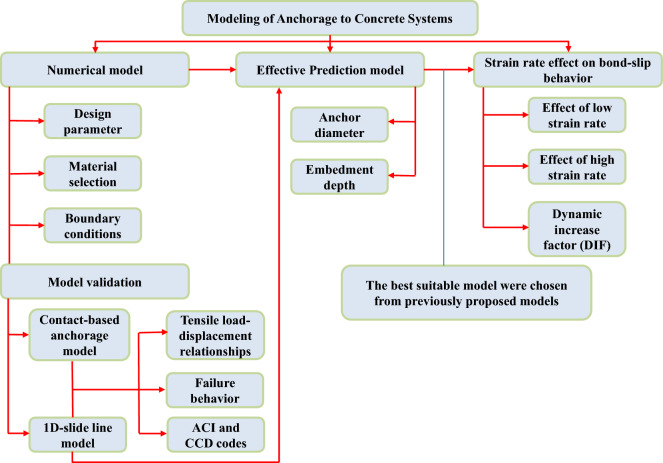
Flow chart for the methodology of this study.

Therefore, the main purpose of this study is to propose an effective model to predict the pull-out strength of a cast-in-place anchor based on the 1D-slide line model. Firstly, a FE method-based anchor model was developed with a contact function and the simulated response was validated with a previous experiment. Next, to reduce the computational time of the contact-based anchor model, simplified anchor models with bond-slip effects between rebars and the surrounding concrete (1D-slide line model) were developed. Then, the bond-slip models proposed from previous experimental studies were investigated to identify which model was appropriate for the simplified model. To maintain structural safety and to reduce or prevent anchorage failure under high-speed dynamic loading, the best proper model was investigated when subjected to various strain rates of loading ranging from the static strain rate of $10^{-5} \mathrm {~s}^{-1}$ to a higher strain rate of $10^{3} \mathrm {~s}^{-1}$ to consider the effect of the design parameters on the capacity of the anchorage. The detailed procedure for this study is shown in Fig. 2.

## Literature review

### Bond-slip model

The structural responses of RC structures were dramatically affected by the bond-slip effects between steel reinforcing bars and concrete^37–39^. 1D-slide line models were used by Shi et al.^40^ to simulate pull-out responses in RC beam elements and the responses were validated with experimental findings. To investigate the bond properties of bars in the post-yield regime, Shima et al.^41^ performed pull-out tests on bars with an equal embedment length, and one of their test specimens was used to perform a FE analysis.

Utilizing the experimental data from the previous research works, it was determined that the proposed model in the FE analysis could accurately represent the bond-slip behavior of bars in RC structures. The bond stress of reinforcing bars is greatly influenced by the concrete strength and reinforcement geometry, such as the rib face angle, spacing and height. Several studies have also been carried out, and various empirical equations have been proposed based on the test results, as shown in Table 1.

**Table 1:** Bond strength equations proposed by previous researchers.

Authors	Equation	Note
CEB–FIP^7^	$\tau _{\max } = 2.5\sqrt{{{f}_{c}}^{'}}$	
Eligehausen et al.^8^	$\tau _{\max } = (20 - d/4)\sqrt{{{f}_{c}}^{'}/30}$	
Esfahani et al.^9^	$\tau _{\max } = 8.6\frac{{c/d + 0.5}}{{c/d + 5.5}}{f_t}$	${f_t} = 0.55\sqrt{{{{f}_{c}}^{'}}}$, ${{f}_{c}}^{'} \ge 50$ MPa
Tepfers^10^	${\tau _{\max }} = (1.53\frac{c}{d} + 0.36){f_t}$	${f_t} = 0.55\sqrt{{{f}_{c}}^{'}}$
Hadi^11^	${\tau _{\max }} = 0.083045\sqrt{{{f}_{c}}^{'}} \left[ {22.8 - 0.208\left( {\frac{c}{d}} \right) - 38.212\left( {\frac{d}{{{h_{ef}}}}} \right) } \right]$	${{{f}_{c}}^{'}} \ge 50$ MPa
Orangun et al.^12,13^	${\tau _{\max }} = (0.1 + 0.25\frac{c}{d} + 4.15\frac{d}{{{h_{ef}}}})\sqrt{{{f}_{c}}^{'}}$	

The CEB–FIP model code^7^ only investigated the effect of the compressive strength of concrete to predict the bond stress of the pull-out test. Eligehausen et al.^8^ investigated pull-out tests with headed studs embedded in large concrete blocks. The embedment depth was varied from 130 to 520 mm and a concrete block with dimensions such as a length of 4 m, width of 2 m and thickness of 0.6 m. The authors proposed the formula for the pull-out test considering the effect of parameters such as rebar diameter and the compressive strength of the concrete.

On the other hand, Esfahani et al.^9^ investigated 284 available tests of normal-strength concrete and high-strength concrete. Tepfers^10^ carried out a pull-out test with concrete with a compressive strength of 24.6 MPa, reinforcing bar diameters from 16 to 25 mm, and vertical concrete cover thickness from 12 to 90 mm. The authors classified the bond behaviors between the reinforcement and concrete into elastic, plastic stages, and presented these behaviors as functions of the tensile strength of the concrete, the concrete cover thickness, and the diameter of the steel reinforcement.

Hadi^11^ studied the bond of high strength concrete using fourteen pull-out tests. The compressive strength of the concrete was 70 MPa and the tensile strength of the steel was 500 MPa. Bar diameters were investigated from 12 to 36 mm. The author proposed an equation for calculating the bond stress depending on parameters such as concrete cover, rebar diameter, embedded depth of rebar, and the compressive strength of the concrete.

Orangun et al.^12,13^ investigated the effect of more parameters while proposing an equation from a nonlinear regression analysis of the test results of beams with lap splices. This reflected the effect of concrete cover, rebar diameter, embedded depth of rebar, and the compressive strength of the concrete and moment gradient on the strength of lap splices.

**Figure 3:**
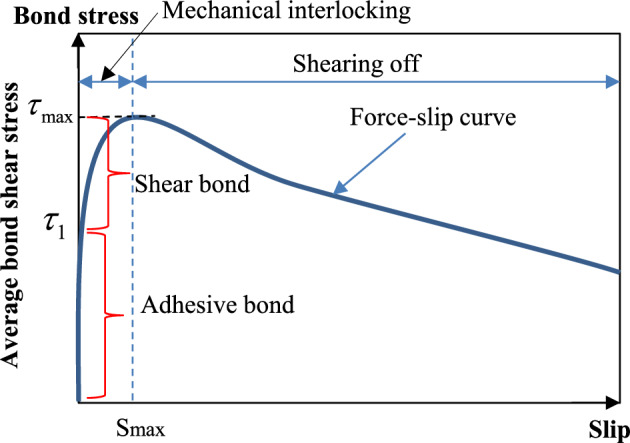
Bond shear stress-slip relationship from a typical pull-out test^37–39^.

A typical average bond stress and slip curve was obtained from their pull-out tests as shown in Fig. 3. As shown in this figure, there is no slip between the steel bar and concrete as the average bond stress grows from zero to $\tau _1$ (adhesive bond). This is because adhesion in this stage is the primary mechanism of stress transfer between the concrete and steel bars. The mechanical contact concentrated at the faces of the ribs dominates the transfer forces as the bond shear stress rises, resulting in slip between the two materials. The bond stress rises to its maximum value $\tau _{\max }$ (shear bond) before the concrete close to the ribs cracks.

The followings should be noted: $\tau _{\max }$ is bond strength, *c* is the concrete cover thickness, *d* is the diameter of the rebar, $h_{ef}$ is the embedded depth of the rebar, ${{f}_{c}^{'}}$ is the compressive strength of the concrete, $f_t$ is the tensile strength of the concrete.

### Finite element based modeling method

There are many FEM-based programs that can be used to study a variety of interactions between rebar and concrete under different loads. Currently, there are two widely used modeling methods, (1) merging nodes between anchor and surrounding concrete meshes and (2) implementing contact option (e.g., the SURFACE_TO_SURFACE_CONTACT option in LS-DYNA) between the rebar and surrounding concrete meshes.

For the merging node approach, Sartipi et al.^42^ investigated the behavior of post-installed adhesive anchoring systems under tensile loading by merging the nodes between anchor, adhesive and concrete to create contact between them. Because this method uses mesh nodes to create interaction between anchor and concrete, this process is arduous as it requires extra effort in meshing. Thus, some mesh distortions may be found in the analysis process. As a result, this method cannot accurately present the failure behavior between anchor and concrete.

To implement contact options (contact-based model), Ahmed et al.^43,44^ used the contact function to simulate interaction between anchor and concrete and investigate the tensile behavior of the anchorage systems, including ultimate force and displacement capacity, load-displacement behavior and the failure models of the undercut and adhesive anchors under different strain loading rates, ranging from $10^{-5} \mathrm {~s}^{-1}$ to $10^{3} \mathrm {~s}^{-1}$. Ahmed et al.^45^ also simulated the shear behavior of cast-in-place anchorage to concrete systems by using contact-based method.

The contact-based method is the most reliable method for modeling the bond between anchor and concrete, as well as predicting the failure behavior between anchor and concrete. However, this modeling method is time consuming because of the contact function between the anchor and surrounding concrete meshes. As an alternative, the authors propose an effective modeling method that balances computational time and accuracy, using a bond-slip modeling method, because the bond slip relationship is crucial to understanding the large-scale mechanical characteristics of composite materials and structures. In this work, an analytical model is created to determine the bond-slip relationship at the anchor-substrate concrete contact (interface).

## Development and validation of numerical model

### Development of FE model

#### Previous experimental studies

**Figure 4:**
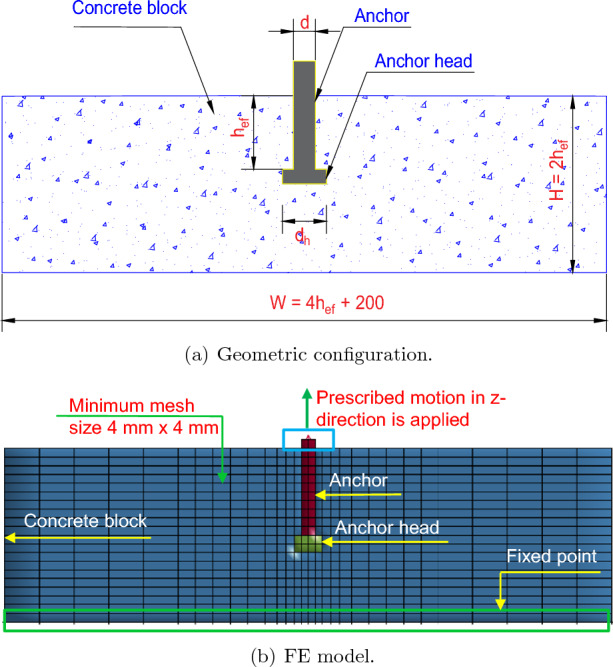
The geometric configuration and FE model for the cast-in-place anchor.

Cast-in-place anchors were the subject of experimental studies by Eligehausen et al.^46^. The main goal of the study was to evaluate the headed anchor bolts’ capacity to support an axial tension load on a concrete cone. Test specimens with different embedment depths of 50 mm, 150 mm, and 450 mm were used. The concrete block size was (${4h}_{ef} + 200$)mm $\times$ (${4h}_{ef} + 200$)mm $\times$ (${2h}_{ef}$)mm. The geometric configuration for the cast-in-place anchor is as shown in Fig. 4a. The circular support’s diameters in this experiment were scaled according to the embedment depth, while the concrete’s physical characteristics were maintained at a constant level. A consistent spacing of 100 mm was maintained between the support reaction and the member’s outside edges. Concrete of essentially the same quality and with the requisite cube strength ${{f}_{c}^{'} = 31}$ MPa was used to construct each example. The dimensions and strength of the specimens are shown in Table 2.

The followings should be noted: ${h}_{ef}$ is the embedded depth of the anchor, *d* is the diameter of the anchor, ${{f}_{y}}$ is the yield strength of the anchor, *W* is the width of the concrete block, *H* is the height of concrete block, ${{f}_{c}}^{'}$ is the compressive strength of the concrete block.

**Table 2:** Dimensions and strength of test specimens from Eligehausen et al.^46^.

Size	Cast-in-place anchor			Concrete block		
	${h}_{ef}$ (mm)	*d* (mm)	${{f}_{y}}$ (MPa)	*W* (mm)	*H* (mm)	${{f}_{c}}^{'}$ (MPa)
Small	50	8	896	400	100	31
Medium	150	24	896	800	300	31
Large	450	72	896	2000	900	31

#### Development of FE model

The ultimate tensile load was derived from the FE analysis. Then, the experimental results published in the literature were compared to validate the FE model. The numerical model was validated using two FE models that represented the test specimens used by Eligehausen et al.^46^. The embedment depths of the steel anchors were 50 mm and 150 mm, respectively, and the concrete block size was (${4h}_{ef} + 200$)mm $\times$ (${4h}_{ef} + 200$)mm $\times$ (${2h}_{ef}$)mm.

The explicit commercial FE code LS-DYNA (LSTC, 2014)^47^ was used to analyze how cast-in-place anchors embedded in concrete behaved under tensile loading. The friction of the cast-in-place anchor on the concrete and the anchor plate was considered. The anchor head plays a significant part in the cast-in-place anchor’s ability to withstand applied loads. Figure 4b depicts the FE model for the cast-in-place anchor. Eight-noded hexahedron solid elements were used to model the anchor and concrete block.

The solid elements for the anchor and concrete block were formulated using a constant stress. There are two different methods that consider interaction between the steel anchor and the concrete, either CONTACT_AUTOMATIC_SURFACE_TO_SURFACE (contact function with friction) or CONTACT_1D (1D-slide line model). The concrete block was modelled using the Karagozian and Case Concrete model (MAT_072R3)^48^ while the steel anchor was modelled using the PIECEWISE_LINEAR_PLASTICITY model representing bilinear material behavior.

### Validation of the contact-based anchorage model

The FE software (LS-DYNA) offers several different contact options. Generally, there are two types of surface contact: automatic contacts and non-automatic contacts. According to LSTC (2014)^47^, the non-automatic contacts function can be utilized for implicit analysis whereas the automated contacts function are suitable for explicit analysis. Automatic contacts can notice penetration occurring from both side of the element as considering the thickness of the element.

**Figure 5:**
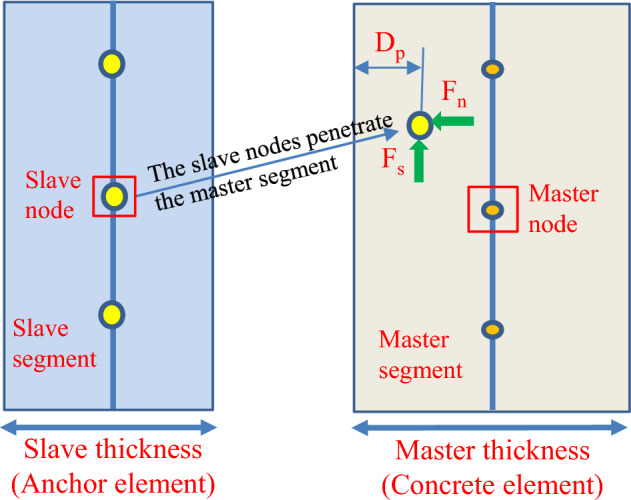
The slave nodes penetrate the master segment in the contact-based anchorage models.

The contact interactions between the anchor, anchor head and concrete block were defined in the current investigation using the CONTACT_AUTOMATIC_SURFACE_TO_SURFACE function. The penetration depth ($D_p$) was used to determine if a slave node had penetrated the master segment, as shown in Fig. 5. The master segment nodes are subjected to a response force such that the combined force acting on the master nodes is equivalent to the force acting on the slave nodes. As a result, the slave node is influenced by the normal force ($F_n$) as well as the friction force ($F_s$), as illustrated in Eqs. (1–2). The stiffness and friction ratio of elements are important parameters directly affecting the normal force and the friction force. Thus, selecting the appropriate element stiffness and friction ratio will enhance the accuracy of the results in the numerical simulation.

1 $$\begin{aligned} F_{n}= & {} \textrm{k} \times D_{p} \end{aligned}$$

2 $$\begin{aligned} F_{s}= & {} \mathrm {\mu }\times F_{n} \end{aligned}$$

where $F_n$ is the normal force; $F_s$ is the friction force; k is the stiffness of element; and $\mu$ is the friction ratio

**Figure 6:**
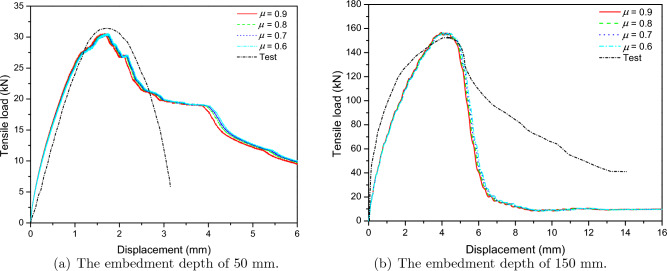
Predict load-displacement curve for cast-in-place anchor based on the contact-based anchorage models.

Figure 6 presents a comparison of tensile load-displacement results from the experimental results by Eligehausen et al.^46^ and the FE analysis for anchor embedment depths of 50 mm and 150 mm. The anchor model was investigated for four friction ratio scenarios, $\mu$ = 0.6–0.9. The friction ratio did not significantly affect the tensile load-displacement relationship, as shown in Fig. 6. The ultimate tensile loads obtained from the FE analysis were found to be 2.82% and 2.44% different, as compared to the experimental results for the 8 mm and 24 mm diameter anchors, respectively. The initial stiffnesses of the concrete anchorage system were 2.62% and 7.2% different as compared with the test results for 50 mm and 150 mm embedded depth, respectively, as shown in Table 3.

**Table 3:** The parameters of the hysteresis diagram of the contact-based anchorage models.

Embedded depth (mm)	Model	Initial stiffness (kN/mm)		Maximum strength (kN)	
		Value	Error (%)	Value	Error (%)
50	Test	18.31	–	31.52	–
	Numerical	17.83	− 2.62	30.63	− 2.82
150	Test	35.84	–	153.04	–
	Numerical	38.42	7.2	156.77	2.44

**Figure 7:**
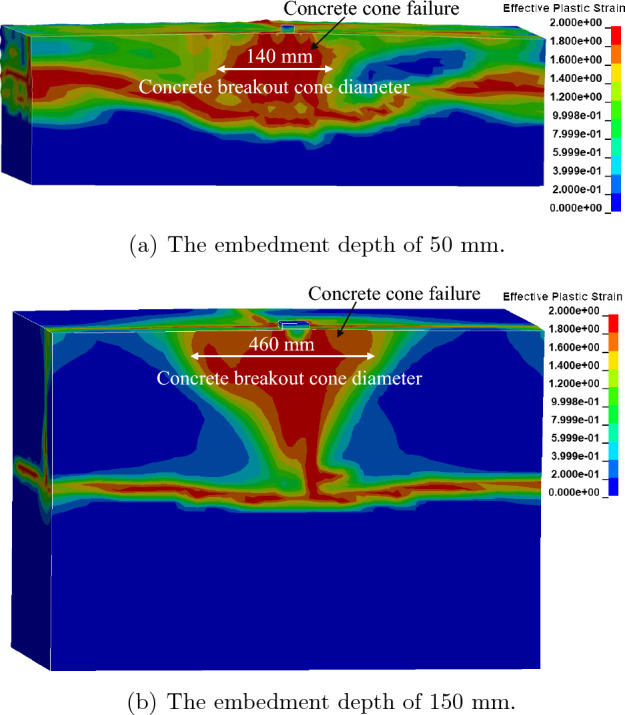
Failure behavior for a cast-in-place anchor based on the contact-based anchorage models.

The FE analysis revealed a concrete cone breakout failure mechanism, as shown in Fig. 7, which is comparable to the results of the experimental results obtained by Eligehausen et al.^46^. Based on the FE analyses, concrete breakout cone diameters of 140 mm and 460 mm were found for 8 mm and 24 mm diameter anchors with 50 mm and 150 mm embedment depths, respectively. The concrete cone breakout diameter, measured from the previous experiment to be less than ${4h}_{ef}$, was in good agreement with the experimental results (200 mm and 600 mm for the 8 mm and 24 mm diameter anchors, respectively).

Although the contact-based anchorage model was able to reasonably predict the tensile load-displacement as well as the failure behavior, when compared to the test data, the analysis of contact-based anchorage models is time-consuming, due to the complicated definition of the contact function between the anchor element and the concrete cover. Therefore, the development of a more simplified model is needed to save analytical time but still ensure reasonable results.

Such a modeling approach can be found in the 1D-slide line model in LS-DYNA. It should be noted that modeling methods similar to the 1D-slide line model in other FE-based simulation tools can be effective.

### Validation of 1D-slide line models

**Figure 8:**
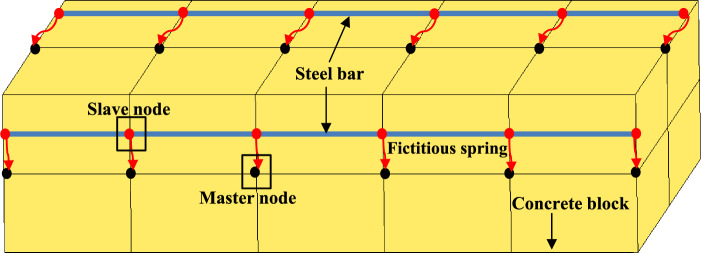
The contact between master and slave nodes in 1D models.

Because this study primarily investigated the effect of interaction between anchor and concrete blocks, the contact interactions between the concrete and anchor head for the cast-in-place anchors system were similarly defined with Contact-based anchorage model by using the CONTACT_AUTOMATIC_SURFACE_TO_SURFACE function. Anchor can slide along a string of concrete nodes using the 1D-slide line model, known in LS-DYNA as CONTACT_1D. It is used to simulate the bond-slip behavior between steel bar and concrete in RC members. In this model, a master line of nodes embedded in the solid mesh that models the concrete matrix forces the slave node of a string of beam or truss elements that models the rebar to slide along it. Fictitious springs are introduced between the slave nodes and their projections across the master lines. Figure 8 illustrates how these springs produce internal forces along the rebar that are proportional to the separation between slave nodes and master lines. By defining the bond shear modulus ($G_s$), maximum elastic slips ($s_{max}$), and the damage curve exponential coefficient ($h_{dmg}$)^40,49^, these 1D-slide line models can simulate the bond-slip effects.

The bond between concrete and steel bars was assumed to be elastic-perfect-plastic when damage accumulation is not taken into account. When damage accumulation is taken into account, the bond shear stress will decay exponentially with the increase in plastic slippage in the plastic range. The constitutive relation between shear stress and slip is represented in Eq. (3).

3 $$\begin{aligned} \tau = {\left\{ \begin{array}{ll}G_{s} s, &{} s \le s_{\max } \\ \tau _{\max } e^{-h_{\textrm{dmg}} D}, &{} s>s_{\max }\end{array}\right. } \end{aligned}$$

where $G_s$ is the bond shear modulus which is the slope of the bond stress-slip curve at the point when the bond stress equals $\tau _1$ (as shown in Fig. 3). The value of $G_s$ ranges from 9.5 to 80.4 MPa/mm obtained experiments are used to define the parameters for the 1D- slide line model; $h_{dmg}$ is the damage curve exponential coefficient which is between 0.05 and 0.24 obtained experiments are used to define the parameters for the 1D- slide line model. It will be adjusted to best fit with the results; $s_{max}$ is the maximum elastic slip, $s_{max}= \tau _{max}/{G_s}$ ; and *D* is the damage parameter defined as the sum of the absolute values of the plastic displacement increments $\Delta \textrm{s}_p$, $\textrm{D}_{{\mathfrak {n}}+1}=\textrm{D}_{{\mathfrak {n}}}+\Delta \textrm{s}_{{\textbf{u}}}$; $\tau _{max}$ is bond strength proposed by previous studies as shown in Table 1^7–13,40,49^.

**Table 4:** The parameters of the hysteresis diagram of the 1D-slide line models.

Embedded depth (mm)	Model	Displacement (mm)		Initial stiffness (kN/mm)		Maximum strength (kN)	
		Value	Error (%)	Value	Error (%)	Value	Error (%)
50	Test	1.68	–	18.31	–	31.52	–
	Bond-slip	2.01	19.05	15.78	− 13.82	31.9	1.20
	Perfect bond	2.96	76.19	12.27	− 32.9	32.10	1.84
150	Test	4.43	–	35.84	–	153.04	–
	Bond-slip	3.80	− 14.22	40.37	12.64	155.8	1.81
	Perfect bond	5.52	24.60	30.73	− 14.26	154.92	1.23

The CEB–FIP model code was used to describe the tensile load-displacement performance in the FE models^50^. According to the bonding conditions (poor and good conditions) and failure modes (such as splitting or pull-out failure modes), the model code describes the relationship between tensile load and displacement (e.g., perfect bond or bond-slip). Constricting forces in relation to the concrete cover and anchorage details can be used to assess the bonding state. There are two scenarios to be considered in the interaction between anchor and concrete, perfect bond and bond-slip. The bond-slip is the relative displacement of concrete and reinforcing material. The term “bond of reinforcement” refers to the ability of the concrete and a reinforcing bar to transfer stress.

**Figure 9:**
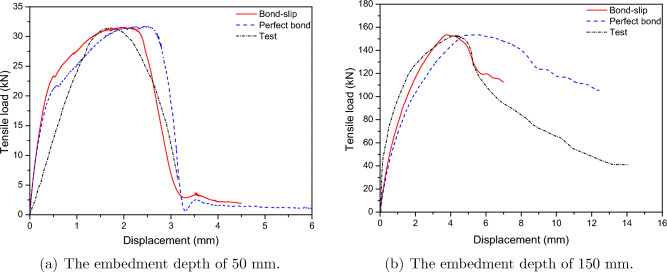
Predicted load-displacement curve for cast-in-place anchor based on the 1D-slide line models.

In an RC construction, a crucial mechanism activates the composite action between concrete and steel. The 1D-slide line model in LS-DYNA and the model code’s tensile displacement relationships are compared in Fig. 9. When the bond-slip effect is taken into consideration using a 1D-slide line model, a better prediction of the model’s reaction can be developed. Although the predicted tensile load was very close to the test data, there was a difference in displacement between the FE model’s result and the test when the bond slip was not taken into account, as shown in Table 4. The bond-slip model predicted less displacement compared to the perfect bond model. The errors in the maximum strength between the experiment and simulation with the bond-slip model and perfect bond model were 1.2% and 1.84%, respectively, for embedded depths of 50 mm, and 1.81% and 1.23%, respectively, for embedded depths of 150 mm. Moreover, the errors of the maximum displacement of bond-slip models were 2.01% and 3.8% for embedded depths of 50 mm and 150 mm, respectively, as well as 2.96% and 5.52% for embedded depths of 50 mm and 150 mm, respectively, for the perfect bond models.

**Figure 10:**
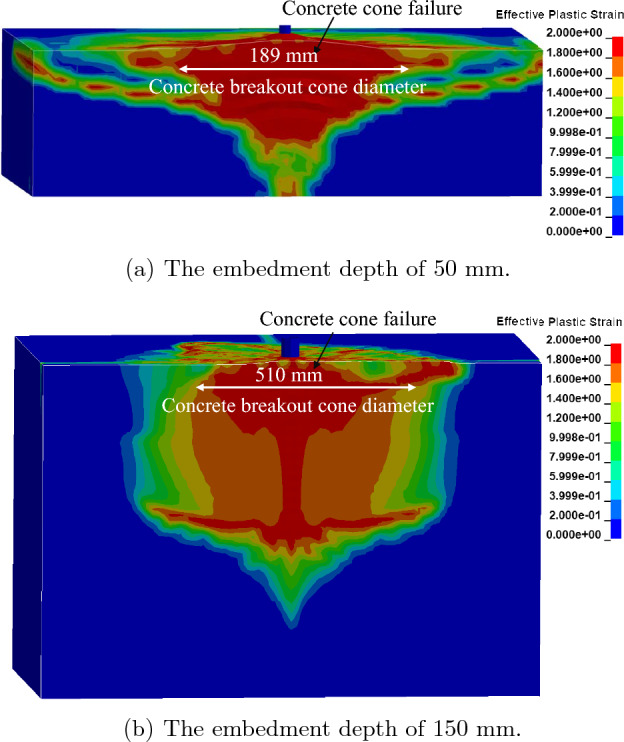
Failure behavior for cast-in-place anchor based on the 1D-slide line models.

The FE analysis revealed a concrete cone breakout failure mechanism, as shown in Fig. 10, which was comparable to the experimental results obtained by Eligehausen et al.^46^. For 8 mm and 24 mm diameter anchors with 50 mm and 150 mm embedment depths, respectively, the FE analysis obtained concrete breakout cone diameters of 189 mm and 510 mm. The experimental findings by Eligehausen et al., who indicated that the concrete cone breakout diameter was less than ${4h}_{ef}$ (200 mm and 600 mm for the 8 mm and 24 mm diameter anchors, respectively), were in good agreement compared to the FE simulation with the 1D-slide line model.

**Figure 11:**
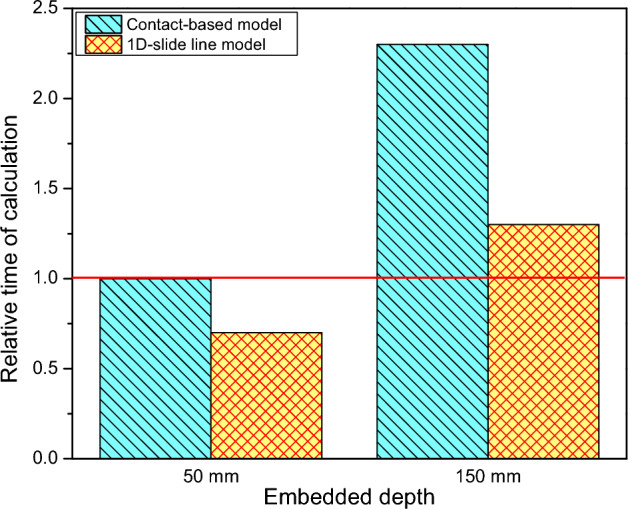
Relative time of calculation versus modeling methods.

Figure 11 shows the computational time between the contact-based model and the 1D-slide line model. The 1D-slide line model was more effective computation time than the contact-based model. It took at least 0.7 times and 0.5 times for mesh size 50 and 150 mm, respectively, as simulating with the 1D-slide line model compared with the contact-based model. Therefore, the 1D-slide line model was an effective method that balances computational time and accuracy.

## Effective prediction model

### Proposed effective prediction models

**Figure 12:**
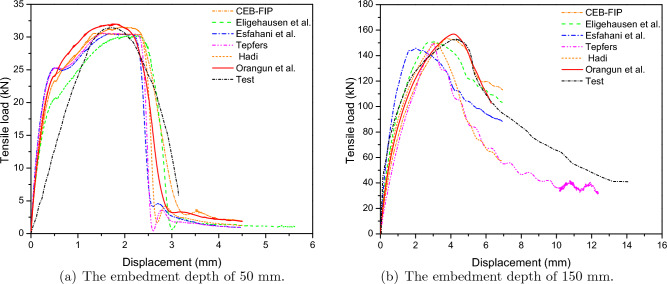
Predicted load-displacement curves of various 1D-slide line models.

Because the 1D-slide line model was effective for predicting the response of the anchor model, as well as minimizing the analytical time, this model will be investigated using various models to find the best suitable model. The prediction of tensile load-displacement curves with various models included experimental results and FE models based on different previous theories, such as CEB–FIP^7^, Eligehausen et al.^8^, Esfahaniet et al.^9^, Tefers^10^, Hadi^11^, and Orangun et al.^12,13^ as shown in Fig. 12. Overall, the initial stiffness of all models was overestimated compared to the test results for two cases, with embedded depths of 50 mm and 150 mm. The maximum difference was 26.87% for the Eligehausen model, and the minimum difference was 2.62% for the Orangun model with the 8 mm diameter anchor. Similarly, the maximum and minimum difference in initial stiffness was 100.39% and 11.16%, for the Esfahani and Orangun models, respectively, for the 24 mm diameter anchor.

On the other hand, the closest displacement compared to the test belonged to the Orangun model with 4.76% and 11.51% for the embedded depth of 50 mm and embedded depth of 150 mm, respectively. The maximum strength of all models did not significantly change as compared to the experiment, as shown in Tables 5 and 6. Because the 1D-slide line models primarily focused on the general bond-slip behavior between the anchor and concrete, and they were limited to considering the surface bond between the anchor and concrete, the 1D-slide line models were less effective in predicting the interaction between the anchor and concrete for models with lower embedded depths.

**Figure 13:**
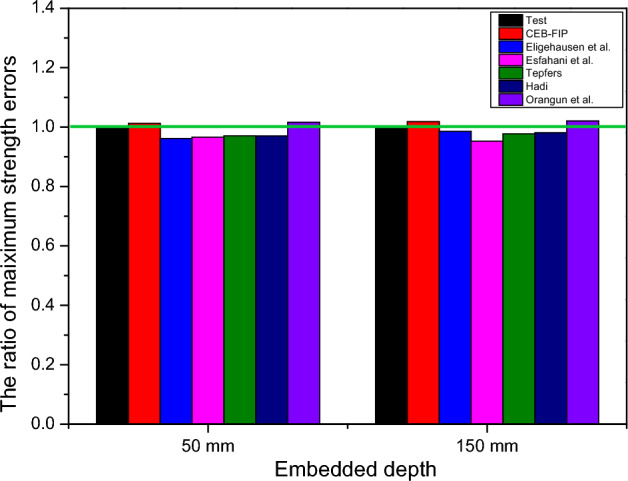
A comparison of strength reduction ratios as tested.

**Figure 14:**
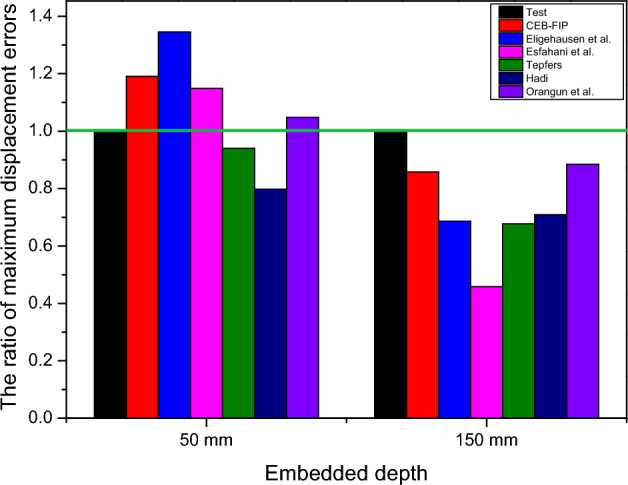
A comparison of displacement reduction ratios as tested.

The ratio of maximum strength errors and the ratio of maximum displacement errors of the 1D-slide line model between the experimental and simulated responses are shown in Figs. 13 and 14. The Orangun and CEB–FIP models had the best predictions for the ratio of maximum strength errors, with 1.01 and 1.02, respectively, for embedded depths of 50 mm, and 1.02 and 1.03, respectively, for embedded depths of 150 mm. Moreover, the Orangun model had the closest prediction for the ratio of maximum displacement errors, with 1.05 and 0.89 for embedded depths of 50 mm and 150 mm, respectively, as compared to the experimental results.

Based on this investigation, the Orangun model was found to produce better agreement than the other models. However, the CEB–FIP model also had reasonable predictions for the relationship between the tensile load-displacement curve, with 1.2% and 19.05% for the maximum strength and displacement of an embedded depth of 50 mm, and 1.81% and 3.8% for the maximum strength and displacement of an embedded depth of 150 mm. Thus, the proper 1D-slide line models selected to simulate the interaction between the anchor and concrete were the CEB–FIP and Orangun models.

**Table 5:** The parameters of an anchor with an embedded depth of 50mm.

Models	Displacement (mm)		Initial stiffness (kN/mm)		Maximum strength (kN)	
	Value	Error (%)	Value	Error (%)	Value	Error (%)
Test	1.68	–	18.31	–	31.52	–
CEB–FIP	2.01	19.05	15.78	− 13.82	31.90	1.20
Eligehausen	2.26	34.52	13.39	− 26.87	30.30	− 3.87
Esfahani	1.93	14.88	15.78	− 13.82	30.45	− 3.39
Tepfers	1.58	− 5.95	19.24	5.08	30.59	− 2.95
Hadi	1.34	− 20.24	22.82	24.63	30.58	− 2.98
Orangun	1.76	4.76	18.79	2.62	32.01	1.55

**Table 6:** The parameters of an anchor with the an embedded depth of 150mm.

Models	Displacement (mm)		Initial stiffness (kN/mm)		Maximum strength (kN)	
	Value	Error (%)	Value	Error (%)	Value	Error (%)
Test	4.43	–	35.84	–	153.04	–
CEB–FIP	3.80	− 14.22	40.37	12.64	155.80	1.81
Eligehausen	3.04	− 31.38	49.60	38.40	150.78	− 1.48
Esfahani	2.03	− 54.18	71.82	100.39	145.79	− 4.74
Tepfers	3.01	− 32.28	49.85	39.09	149.56	− 2.27
Hadi	3.14	− 29.12	47.78	33.31	150.03	− 1.97
Orangun	3.92	− 11.51	39.84	11.16	156.17	2.05

### Comparison of FE results with ACI and CCD design codes

As mentioned above, the two FE models investigated had embedment depths of 50 mm and 150 mm, and the concrete block size was (${4h}_{ef} + 200$)mm $\times$ (${4h}_{ef} + 200$)mm $\times$ (${2h}_{ef}$)mm. Results of the FE analysis for ultimate tensile loads were compared to those from the Concrete Capacity Design (CCD) and American Concrete Institute (ACI) 318 methods and are shown in Table 7. In this part, the 1D-slide line model with the Orangun model was examined. The bond-slip model proposed by Orangun et al.^12,13^ simulates bond-slip behavior with minimum errors among the considered models referenced as the ’best fit model’ Eqs. (4)-(5) and can be used to calculate the ultimate tensile load of the cast-in-place anchors in accordance with the ACI method^51^ and CCD method^52^.

4 $$\begin{aligned} N_{c b}=\left( A_{N c} / A_{N c o}\right) \times \psi _{e d, N} \times \psi _{c, N} \times \psi _{c p, N} \times N_{b} \end{aligned}$$

where $N_{cb}$ is nominal concrete breakout strength in tension of a single anchor; $A_{Nc}$ is projected concrete failure area of a single anchor; $A_{Nco}$ is projected concrete failure area of a single anchor for calculating of strength in tension if not limited by edge distance or spacing; $N_b$ is basic concrete breakout strength in tension of a single anchor in cracked concrete.

5 $$\begin{aligned} N_{b}=k_{c} \sqrt{f_{c}^{\prime }} h_{ef}^{1.5} \end{aligned}$$

where ${f_c}^{'}$ represents nominal concrete strength, $h_{ef}$ is the effective embedment depth, and $k_c$ is the coefficient for the cast-in anchors equal to 15

**Figure 15:**
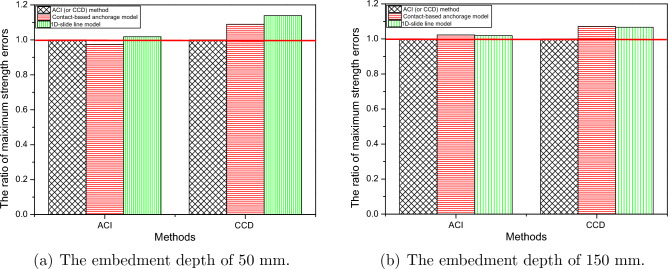
The ratio of maximum strength errors between FE simulation and code-defined method as compared to ACI and CDD methods.

According to Table 7, for both the FE analysis and the design approaches, the ultimate tensile load increased with increasing anchor embedment depth. As illustrated in Fig. 15, the 1D-slide line model captures a reasonable prediction as compared to the ACI and CCD approaches. The findings indicate that the FE method approach did not significantly change, as compared with the CCD approach, with 8.93% and 7.12% for embedment depths of 50 mm and 150 mm, respectively. However, the ACI approach and the FE method agreed more closely with 1.78% and 1.88 % for embedment depths of 50 mm and 150 mm, respectively.

**Table 7:** Comparison of ultimate tensile loads obtained from FE model with ACI and CCD.

Methods	Embedded depth (mm)			
	50		150	
	Value (kN)	Error (%)	Value (kN)	Error (%)
ACI	31.45	–	153.29	–
Contact-based anchorage model	30.63	− 2.60	156.77	2.27
1D-slide line model (Orangun model)	32.01	1.78	156.17	1.88
CCD	28.12	–	146.35	–
Contact-based anchorage model	30.63	8.93	156.77	7.12
1D-slide line model (Orangun model)	32.01	13.83	156.17	6.71

## Strain rate effect on bond-slip behavior

**Figure 16:**
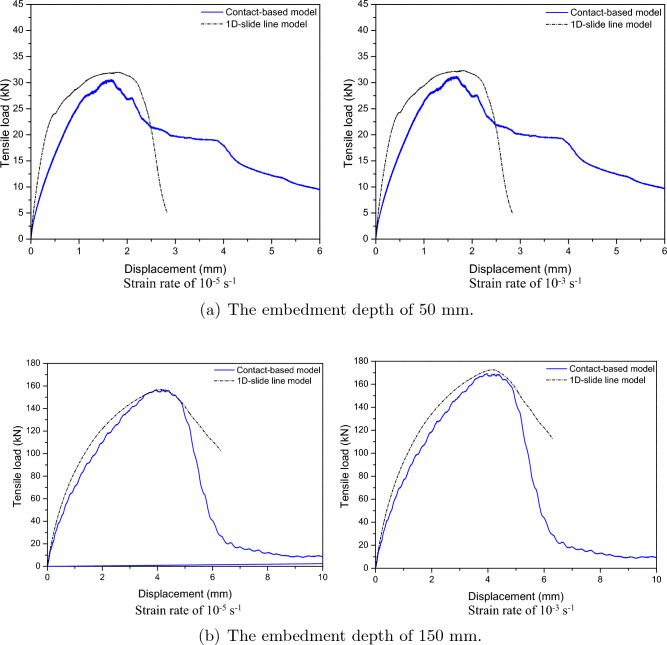
Tensile load-displacement relations between contact-based model and 1D-slide line model at low strain rate.

**Figure 17:**
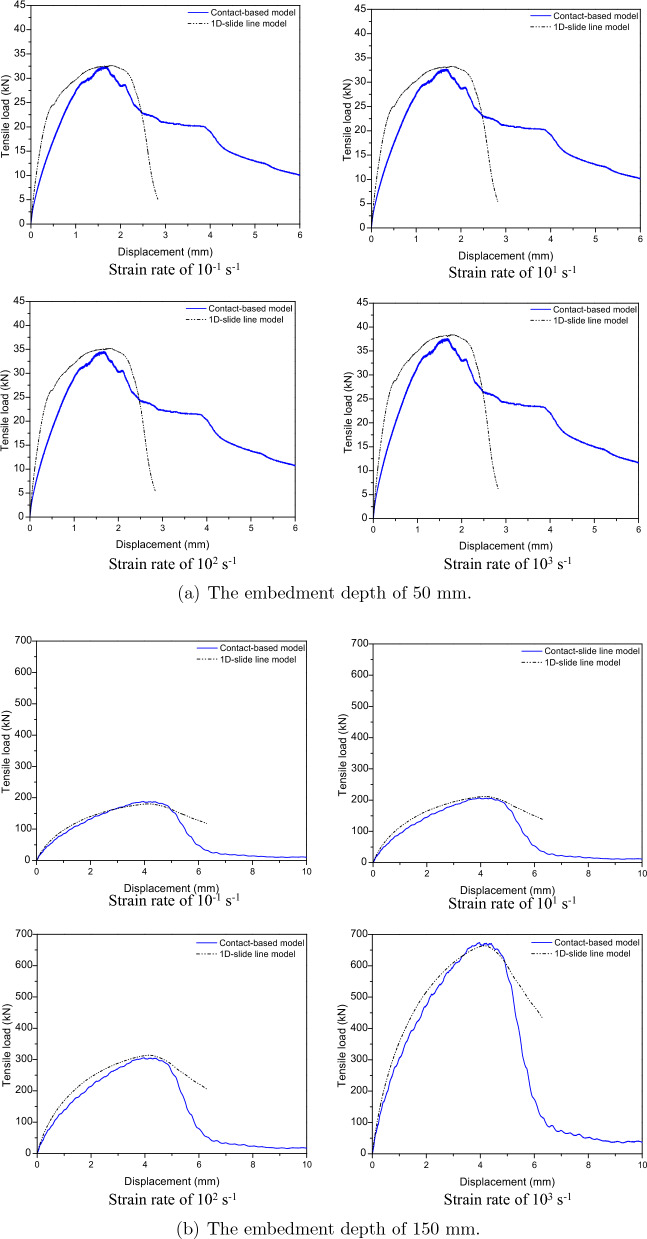
Tensile load-displacement relations between contact-based model and 1D-slide line model at high strain rate.

To validate the effectiveness of 1D-slide line model under strain rate effect, the investigation and comparison between contact-based models and 1D-slide line models under different strain rates is shown in Figs. 16 and 17. From these figures, the 1D-slide line and contact-based models were similar prediction for displacement-tensile load curves behavior of cast-in-place anchorage system with not significant difference about tensile load under different strain rates as shown in Table 8. This demonstrates that the 1D-slide line model is suitable for predicting the response of anchorage system under different strain rates.

The 1D-slide line models with the Orangun model (best-fitted model) were used in this parametric analysis to investigate the effect of different design parameters (embedment depth and anchor diameter) on the behavior of the anchorage system considering strain rate effects. The various models were developed with 8 mm, 12 mm, 16 mm, 20 mm and 24 mm diameter steel anchors and embedment depths of 50 mm, 75 mm, 100 mm and 150 mm. The effect of the design parameters on the anchorage system’s capacity were investigated under various strain loading rates, starting with a low strain rate of $10^{-5} \mathrm {~s}^{-1}$ to a higher strain rate of $10^{3} \mathrm {~s}^{-1}$.

**Table 8:** Comparison of ultimate tensile loads between contact-based model and 1D-slide line model.

$\mathrm {~h_{ef}}$	Strain rate	Contact-based model		1D-slide line model	
	($\mathrm {~s}^{-1}$)	Value (kN)	Errors (%)	Value (kN)	Errors (%)
50 mm	$10^{-5}$	30.6	–	32.01	4.6
	$10^{-3}$	31.21	–	32.33	3.59
	$10^{-1}$	32.44	–	32.65	0.65
	$10^{1}$	32.74	–	33.29	1.7
	$10^{2}$	34.58	–	35.21	1.82
	$10^{3}$	37.64	–	38.41	2.05
150 mm	$10^{-5}$	156.77	–	156.17	0.38
	$10^{-3}$	169.31	–	172.59	1.94
	$10^{-1}$	188.13	–	180.44	4.09
	$10^{1}$	206.94	–	211.82	2.36
	$10^{2}$	305.71	–	313.8	2.65
	$10^{3}$	674.12	–	663.7	1.55

### Modeling approach of strain rate effect

The capacity of concrete constructions is influenced by the amount of applied strain rate. At strain rates between $10^{-6} \mathrm {~s}^{-1}$ and $10^{-5} \mathrm {~s}^{-1}$, static loading can be achieved. Strain rates between $10^{-4} \mathrm {~s}^{-1}$ and $10^{-1} \mathrm {~s}^{-1}$ are produced by low dynamic loading and earthquakes. Impact loading produces a strain rate in the range of $10^{0} \mathrm {~s}^{-1}$ to $10^{1} \mathrm {~s}^{-1}$, whereas blast loading produces a very high strain rate of $10^{2} \mathrm {~s}^{-1}$ to $10^{3} \mathrm {~s}^{-1}$^53^. Varied loading conditions can be applied to concrete structures at various strain rates using various testing equipment. Static loads can be applied using hydraulic testing equipment at strain rates ranging from $10^{-5} \mathrm {~s}^{-1}$ to $10^{-1} \mathrm {~s}^{-1}$. Dynamic loads can be applied using a charpy impact testing machine at a strain rate from $10^{1} \mathrm {~s}^{-1}$ to $10^{3} \mathrm {~s}^{-1}$.

The mechanical characteristics of the steel material are affected by the strain rate. The yield and tensile strength of steel increase as the strain rate increases^35,54,55^, while the Young’s modulus remains constant^54^. This is a result of the steel structure’s deformations and dislocations, which increase under high strain rates^56^. Under low strain rates or quasi-static loads, the deformation of the steel material is almost homogeneous and is influenced by slip and twin plastic deformation mechanisms, which is another mechanism of plastic deformation. Twinning is a surface defect and involves the shearing of an element by a fixed magnitude, based on the characteristics of the element structure. However, where the strains are extremely concentrated along a restricted area, known as the adiabatic shear band, the deformation of the steel material at high strain rate is more complicated. These shear bands operate as the source of cracks that eventually lead to fracture. Several factors, including the chemical makeup of the steel components, strain rate, and heat treatment, have an effect on the formation of the shear band in steel at high strain rates^57^.

The DIF versus strain rate, which is the ratio of dynamic to static strength, is typically used to explain how strain rate affects concrete strengths. A DIF was added to the KCC model to account for the strain rate effect, expressed as follows^58^:

For compressive:

6 $$\begin{aligned} D I F= {\left\{ \begin{array}{ll}\left( {\dot{\varepsilon }} / {\dot{\varepsilon }}_{s c}\right) ^{1.026 \alpha _{s}}, &{} \text{ for } \varepsilon _{s} \le 30 s^{-1} \\ \gamma _{s}\left( {\dot{\varepsilon }} / {\dot{\varepsilon }}_{s c}\right) ^{1 / 3}, &{} \text{ for } \varepsilon _{s}>30 s^{-1}\end{array}\right. } \end{aligned}$$

For tension:

7 $$\begin{aligned} D I F= {\left\{ \begin{array}{ll}\left( {\dot{\varepsilon }} / {\dot{\varepsilon }}_{s t}\right) ^{\delta }, &{} \text{ for } \varepsilon _{s} \le 1.0 \mathrm {~s}^{-1} \\ \beta \left( {\dot{\varepsilon }} / {\dot{\varepsilon }}_{s t}\right) ^{1 / 3}, &{} \text{ for } \varepsilon _{s}>1.0 \mathrm {~s}^{-1}\end{array}\right. } \end{aligned}$$

where ${\dot{\varepsilon }}$ = strain rate in $s^{-1}$ (1/second), ${\dot{\varepsilon }}_{sc} = 30\times 10^{-6}$ $s^{-1}$ for a static strain rate in compression, ${\dot{\varepsilon }}_{st} =10^{-6}$ $s^{-1}$ for a static strain rate in tension, ${{f}_{c}}^{'}$ is static compressive strength of concrete in MPa, $\log \left( \gamma _{s}\right) =6.156 \alpha _{s}-2, \alpha _{s}=1 /\left( 5+0.9 {{f}_{c}}^{'}\right) , \log (\beta )=6 \delta -2$, and $\delta =1 /\left( 1+0.8 {{f}_{c}}^{'}\right)$.

### Tensile load-displacement relationship with strain rate effect

**Figure 18:**
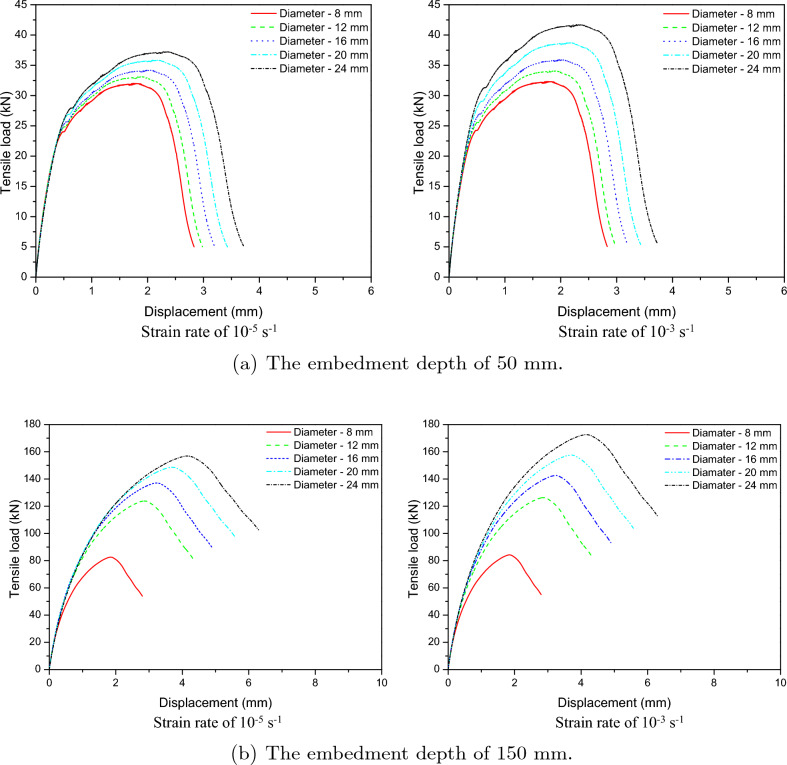
Tensile load-displacement relations at low strain rate.

Numerical models of cast-in-place anchorage to concrete structures were developed in order to examine the tensile behavior of the anchors at low strain rates. To examine the effects of low strain rates on the behavior of cast-in-place anchor systems, strain rates of $10^{-5} \mathrm {~s}^{-1}$ and $10^{-3} \mathrm {~s}^{-1}$ were used for the investigation. Figure 18 shows the load-displacement relations for embedded depths of 50 mm and 150 mm at low strain rates of $10^{-5} \mathrm {~s}^{-1}$ and $10^{-3} \mathrm {~s}^{-1}$. The tensile load increased with displacement as seen in Fig. 18 reaching its maximum value at strain rates of $10^{-5} \mathrm {~s}^{-1}$ and $10^{-3} \mathrm {~s}^{-1}$. The post-peak response demonstrates a decrease in load with an increase in displacement until failure.

The contact area between the cast-in-place anchor and concrete increased with the increase in anchor diameter from 8 to 24 mm, which also increased the anchorage’s tensile strength. This figure shows that the peak loads of diameters from 8 to 12 mm for strain rates of $10^{-5} \mathrm {~s}^{-1}$ and $10^{-3} \mathrm {~s}^{-1}$ were not significantly changed. However, the maximum loads of 16 mm to 24 mm diameters increased as the strain rate changed from $10^{-5} \mathrm {~s}^{-1}$ to $10^{-3} \mathrm {~s}^{-1}$.

**Figure 19:**
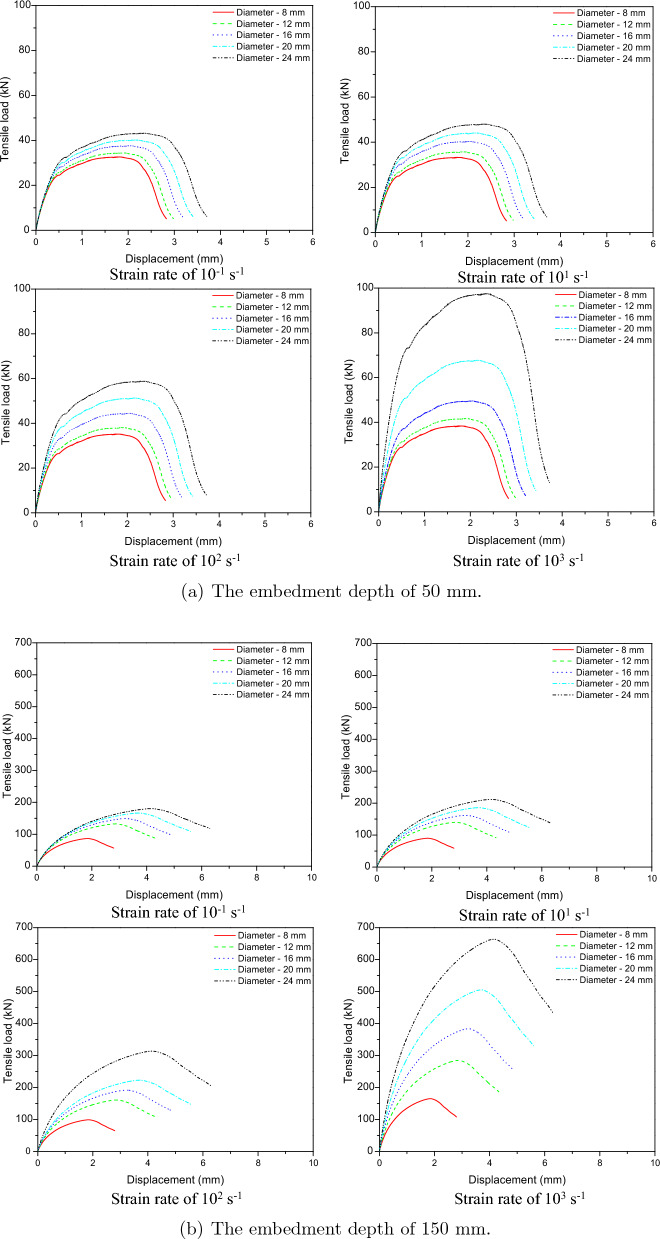
Tensile load-displacement relations at high strain rate.

To determine the effect of high strain rates on the behavior of cast-in-place anchor systems, strain rates of $10^{-1} \mathrm {~s}^{-1}$, $10^{1} \mathrm {~s}^{-1}$, $10^{2} \mathrm {~s}^{-1}$, and $10^{3} \mathrm {~s}^{-1}$ were used. Figure 19 shows load-displacement relations for embedded depths of 50 mm and 150 mm under high strain rates. The ultimate tensile strength increased as the diameter expanded from 8 to 24 mm. The anchoring diameter was significantly affected by the high strain rate. The ultimate tensile of diameter increased significantly from 8 to 24 mm when the strain rate was raised from $10^{1} \mathrm {~s}^{-1}$ to $10^{3} \mathrm {~s}^{-1}$.

**Figure 20:**
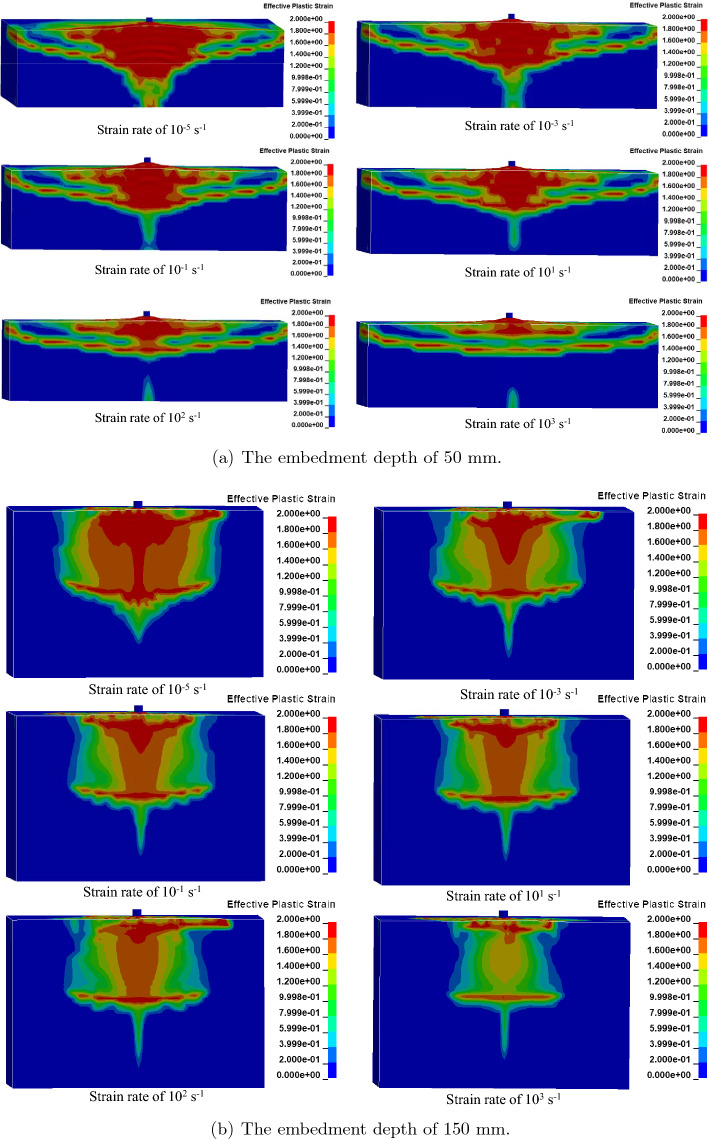
Failure model of cast-in-place anchor at different strain rates.

Figure 20 depicts the failure mode of cast-in-place anchors with varied embedment depths such as 50 mm and 150 mm at strain rates ranging from $10^{-5} \mathrm {~s}^{-1}$ to $10^{3} \mathrm {~s}^{-1}$. Cracks began to emerge above the anchor head early in the loading process, and as time passed, cracks appeared on the top surface of the concrete around the anchor perimeter. Cracks form along the embedment depth and spread to a larger region of the concrete. These cracks subsequently progressed diagonally, resulting in concrete cone breakout failure at strain rates of up to $10^{1} \mathrm {~s}^{-1}$. The increase in the strain rate increased the concrete strength and thus resulting in steel anchor failure was observed at high strain rates of $10^{2} \mathrm {~s}^{-1}$ and $10^{3} \mathrm {~s}^{-1}$.

### Dynamic increase factor (DIF) for embedment depths

The DIF is defined as the dynamic to static strength ratio of a cast-in-place anchor. The effect of DIF on the strength of concrete (tensile and compressive strength) or the strength of rebar (ultimate and yield strength) has been reported by previous research^33–36^. In this part, the effect of DIF on the anchorage to concrete system was investigated in order to predict the response of the cast-in-place anchorage to concrete system, with varying anchor embedment depth from 50 to 150 mm, and anchor diameters from 8 to 24 mm. For comparison with a cast-in-place anchor capacity at higher strain rates, the baseline strain rate of $10^{-5} \mathrm {~s}^{-1}$, which is representative of the static loading rate, was employed.

**Figure 21:**
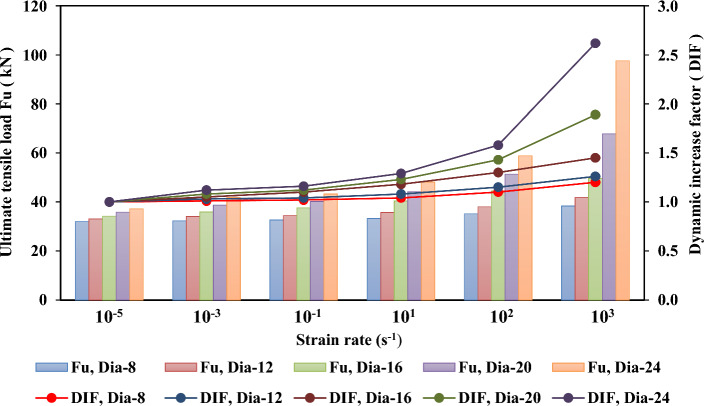
Ultimate tensile load and DIF versus strain rate for the embedded depth of 50 mm.

**Figure 22:**
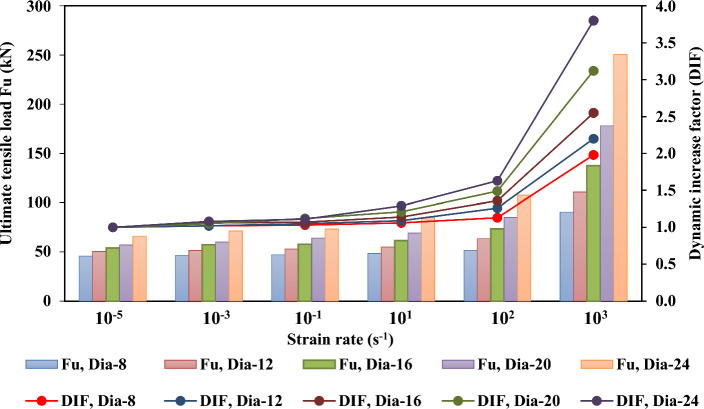
Ultimate tensile load and DIF versus strain rate for the embedded depth of 75 mm.

**Figure 23:**
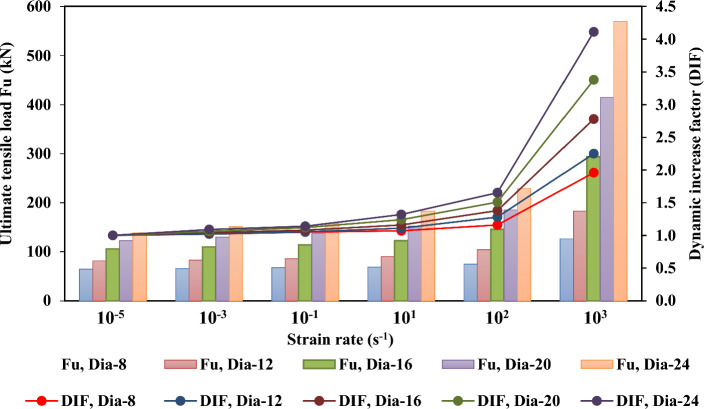
Ultimate tensile load and DIF versus strain rate for the embedded depth of 100 mm.

**Figure 24:**
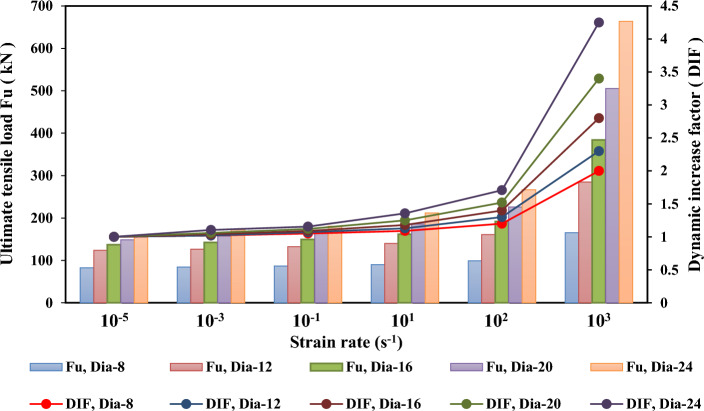
Ultimate tensile load and DIF versus strain rate for the embedded depth of 150 mm.

Figures 21, 22, 23, and 24 show the effect of strain rate on the ultimate tensile load and DIF for anchor embedded depths from 50 to 150 mm with anchor diameters from 8 to 24 mm. The relationship between the ultimate tensile load and the strain rate appears to be bilinear. As illustrated in Figs. 22, 23, and 24 for anchor embedded depths from 75 to 150 mm, the bilinear relationship between the ultimate tensile load and the strain rate was obtained with a sudden change in the slope of DIF versus strain rate curve at strain rate of $10^{2} \mathrm {~s}^{-1}$ where the failure model of concrete cone breakout was observed. The failure model of steel anchor was observed at the highest strain rate of $10^{3} \mathrm {~s}^{-1}$.

For the anchor embedded depth of 50 mm, the slope of DIF versus strain rate curve did not appear for anchor diameters from 8 to 16 mm, as shown Fig. 21. Thus, the relationship between the ultimate tensile load and the strain rate is linear for anchor diameters from 8 to 16 mm. The failure of the steel anchor was the principal failure mode at all strain rates considered. For anchor diameters of 20 mm and 24 mm, the relationship between the ultimate tensile load and the strain rate appears to be bilinear, as a change in slope of the DIF versus strain rate curve appears at a strain rate of $10^{1} \mathrm {~s}^{-1}$ where the failure mode of the concrete cone breakout was observed. The failure mode of the steel anchor was noticed at the higher strain rate from $10^{2} \mathrm {~s}^{-1}$ to $10^{3} \mathrm {~s}^{-1}$.

Table 9 presents the ultimate tensile load ($\mathrm {~F_u}$) and the dynamic increase factor (DIF) of the cast-in-place anchorage to concrete system, with different embedment depths and anchor diameters at strain rates varying from $10^{-5} \mathrm {~s}^{-1}$ to $10^{3} \mathrm {~s}^{-1}$. The ultimate tensile capacity and DIF increased as the strain rates increased. For the anchor embedded depth of 150 mm with an anchor diameter of 24 mm, the ultimate tensile increased from 156.17 to 663.73 kN, and the DIF increased from 1.0 to 4.25 as the strain rate increased from $10^{-5} \mathrm {~s}^{-1}$ to $10^{3} \mathrm {~s}^{-1}$, respectively.

On the other hand, the ultimate tensile capacity and DIF increased as the anchor embedded depths and diameters increased. The ultimate tensile changed from 97.62 to 663.73 kN and the DIF changed from 2.62 to 4.25 as anchor embedded depths increased from 50 to 150 mm, respectively, at a strain rate of $10^{3} \mathrm {~s}^{-1}$. As the diameter of the anchor changed from 8 to 24 mm, with an anchor embedded depth of 150 mm, the ultimate tensile increased from 165.16 to 663.73 kN and the maximum DIF increased from 2.02 to 4.25, respectively, at the highest strain rate of $10^{3} \mathrm {~s}^{-1}$. Thus, increases in the anchor embedded depth and the anchor diameters resulted in increasing ultimate tensile load and DIF at all strain rates investigated.

Finally, the anchor embedded depth was determined to have a significant effect on the ultimate tensile and DIF at a strain rate of $10^{3} \mathrm {~s}^{-1}$. It appeared as a sudden change in the slope of DIF versus strain rate curve at a strain rate of $10^{2} \mathrm {~s}^{-1}$ for all types of anchor diameters. However, the ultimate tensile and maximum DIFs at an anchor embedded depth of 50 mm was not significantly affected by the strain rate effect with anchor diameters of 8 mm, 12 mm and 16 mm, because no slope appeared on the DIF versus strain rate curve, as shown Fig. 21.

**Table 9:** Ultimate tensile load ($\mathrm {~F_u}$) and the dynamic increase factor (DIF) for the cast-in-place anchors at different strain rates.

$\mathrm {~h_{ef}}$ (mm)	Strain rate ($\mathrm {~s}^{-1}$)	Ultimate tensile load ($\mathrm {~F_u}$)					Dynamic increase factor (DIF)				
		d = 8	d = 12	d = 16	d = 20	d = 24	d = 8	d = 12	d = 16	d = 20	d = 24
		mm	mm	mm	mm	mm	mm	mm	mm	mm	mm
50	$10^{-5}$	32.01	33.12	34.22	35.88	37.26	1.00	1.00	1.00	1.00	1.00
75		45.62	50.34	53.95	57.12	65.98	1.00	1.00	1.00	1.00	1.00
100		64.32	81.34	105.65	122.67	138.60	1.00	1.00	1.00	1.00	1.00
150		82.58	123.87	137.08	148.64	156.17	1.00	1.00	1.00	1.00	1.00
50	$10^{-3}$	32.33	34.11	35.93	38.75	41.73	1.01	1.03	1.05	1.08	1.12
75		46.53	51.35	57.19	59.98	71.26	1.02	1.02	1.06	1.05	1.08
100		65.61	82.97	109.88	130.03	151.07	1.02	1.02	1.04	1.06	1.09
150		84.23	126.35	142.57	157.56	172.59	1.01	1.02	1.04	1.06	1.11
50	$10^{-1}$	32.65	34.44	37.64	40.19	43.22	1.02	1.04	1.10	1.12	1.16
75		46.99	52.86	57.73	63.97	73.24	1.03	1.05	1.07	1.12	1.11
100		67.54	85.73	114.10	137.39	158.00	1.05	1.05	1.08	1.12	1.14
150		86.71	132.54	149.42	166.48	180.44	1.05	1.07	1.09	1.12	1.16
50	$10^{1}$	33.29	35.77	40.38	44.13	48.07	1.04	1.08	1.18	1.23	1.29
75		48.36	54.87	61.50	69.12	85.11	1.06	1.09	1.14	1.21	1.29
100		68.82	90.29	122.55	152.11	182.95	1.07	1.11	1.16	1.24	1.32
150		90.01	139.97	161.76	185.81	211.82	1.09	1.13	1.18	1.25	1.36
50	$10^{2}$	35.21	38.09	44.49	51.31	58.87	1.10	1.15	1.30	1.43	1.58
75		51.55	63.43	73.37	85.11	107.55	1.13	1.26	1.36	1.49	1.63
100		74.61	104.12	145.80	185.23	229.11	1.16	1.28	1.38	1.51	1.65
150		99.10	161.03	191.92	225.94	266.73	1.20	1.30	1.40	1.52	1.71
50	$10^{3}$	38.41	41.73	49.62	67.81	97.62	1.20	1.26	1.45	1.89	2.62
75		90.33	110.75	137.57	178.21	250.72	1.98	2.20	2.55	3.12	3.80
100		126.07	183.02	293.71	414.62	569.65	1.96	2.25	2.78	3.38	4.11
150		165.16	284.90	383.83	505.39	663.70	2.02	2.31	2.80	3.42	4.25

## Conclusions

An effective model for predicting the pull-out capacity of cast-in-place anchors was investigated using FE analysis. Cast-in-place anchor diameters of 8 mm to 24 mm with anchor embedded depths of 50 mm and 150 mm were investigated. The main conclusions obtained from the FE analysis of the cast-in-place anchors can be summarized as follows:

1. The contact-based and 1D-slide line models approaches (implementing bond-slip behavior) could reasonably predict the interaction between the cast-in-place anchor and concrete. The 1D-slide line approach with the CEB–FIP model code^7^ and Orangun model^12,13^ were determined to be the proper models to achieve accurate results and save analysis time.
2. The contact-based and 1D-slide line models well predicted the failure behavior of the cast-in place anchorage system by detecting reasonable concrete breakout cone diameter. The 1D-slide line models with the CEB–FIP model code and Orangun model reasonably predicted concrete breakout cone diameters of 166 mm and 510 mm for embedded depths of 50 mm and 150 mm, respectively, less than ${4h_{ef}}$ which was the limited concrete breakout cone diameter reported by Eligehausen et al.^46^.
3. The maximum tensile load was not significantly changed for anchor diameters less than or equal to 12 mm, and slightly increased for anchor diameters more than or equal 16 mm under low strain rate, from $10^{-5} \mathrm {~s}^{-1}$ to $10^{-3} \mathrm {~s}^{-1}$. The ultimate tensile strength significantly increased for anchor diameters of 8 mm or more under high strain rates, from $10^{1} \mathrm {~s}^{-1}$ to $10^{3} \mathrm {~s}^{-1}$. Therefore, the strain rate effect should be considered for anchor diameters more than or equal to 16 mm in cases of low or high strain rates.
4. The relationship between the ultimate tensile load and the strain rate was a bilinear trend for anchor embedded depths from 75 to 150 mm and anchor embedded depth of 50 mm with an anchor diameter of more than 16 mm. The relationship between the ultimate tensile load and the strain rate was a linear trend for an anchor embedded depth of 50 mm with an anchor diameter of less than or equal to 16 mm. The ultimate tensile load and DIF of the cast-in-place anchors increased as the strain rate increased from $10^{-5} \mathrm {~s}^{-1}$ to $10^{3} \mathrm {~s}^{-1}$.
5. The anchor embedded depth had a significant effect on the ultimate tensile and DIF at a strain rate of $10^{3} \mathrm {~s}^{-1}$ (e.g., blast effect). The ultimate tensile and maximum DIF for an anchor embedded at a depth of 50 mm was not significantly affected by the strain rate effect, with anchor diameters from 8 to 16 mm. Thus, the DIF effect should be investigated for anchor embedded depths of more than 50 mm. For an anchor embedded depth of 50 mm, the DIF effect should be considered for anchor diameters of more than 16 mm.
6. The models of cast-in-place anchorage to concrete systems under impact or blast loading are also effective for depicting the exact behavior of cast-in-place anchorage to concrete systems. This study recommends using FE simulations for the DIFs of the anchorage system with respect to the embedment length and anchor diameter. This recommended approach can help more accurately predict the response of the anchorage system under impact or blast loads.

## Data Availability

The datasets used and/or analyzed during the current study available from the corresponding author on reasonable request.

## References

[1] Choi JS, Chin WJ, Yuan TF, Yoon YS (2022). Shear behavior of single cast-in anchor simulating characteristics of bridge bearing anchor. Sci. Rep..

[2] Lee NH, Park KR, Suh YP (2011). Shear behavior of headed anchors with large diameters and deep embedments in concrete. Nucl. Eng. Des..

[3] Qian Zz, Lu Xl, Sheng Mq (2019). Experimental investigation of the tensile capacity for anchor groups with different spacing between cast-in-place headed anchors of high strength and deep embedment. Arabian J. Sci. Eng..

[4] Guo J, Gou Y, Li Z, Guo Q (2023). Experimental research on static and cyclic pullout behavior of cast-in-place anchors with steel sleeve. Structures.

[5] Bang JS, Kwon Y, Ahn JH, Yim HJ (2022). Pull-out behavior evaluation of torque-controlled expansion anchors under various installation conditions of concrete. Case Stud. Constr. Mater..

[6] Delhomme F, Roure T, Arrieta B, Limam A (2015). Tensile behaviour of cast-in-place headed anchors with different embedment depths. Eur. J. Environ. Civ. Eng..

[7] CEB-FIP. *Comite Euro-International du Beton-Federation International de la Pre-contrainte (ceb-fip), Model Code 90 for Concrete Structures*. (ed: Thomas Telford, London, 1993).

[8] Eligehausen, R., & Sawade, G. A fracture mechanics based description of the pull-out behavior of headed studs embedded in concrete (1989).

[9] Esfahani MR, Rangan BV (1998). Bond between normal strength and high-strength concrete (HSC) and reinforcing bars in splices in beams. Struct. J..

[10] Tepfers R (1979). Cracking of concrete cover along anchored deformed reinforcing bars. Mag. Concr. Res..

[11] Hadi MN (2008). Bond of high strength concrete with high strength reinforcing steel. Civ. Eng. J..

[12] Orangun C, Jirsa JO, Breen JE (1975). The Strength of Anchored Bars: A Reevaluation of Test Data on Development Length and Splices.

[13] Orangun C, Jirsa J, Breen J (1977). A reevaulation of test data on development length and splices. J. Proc..

[14] Obayes O, Gad E, Lee J, Pokharel T, Abdouka K (2022). Assessment of the tensile behaviour of cast-in headed anchors with void formers in early age concrete. Constr. Build. Mater..

[15] Karmokar T, Mohyeddin A, Lee J, Paraskeva T (2021). Concrete cone failure of single cast-in anchors under tensile loading-a literature review. Eng. Struct..

[16] Nilforoush R, Nilsson M, Elfgren L (2017). Experimental evaluation of tensile behaviour of single cast-in-place anchor bolts in plain and steel fibre-reinforced normal-and high-strength concrete. Eng. Struct..

[17] Mousavi Siamakani SY, Sahamitmongkol R (2022). Post-installed anchors in concrete strengthened by post-installed reinforcement under tensile load. Eur. J. Environ. Civ. Eng..

[18] Al Saeab, L. T. A. *Finite Element Modelling of Anchorage to Concrete Systems at Different Strain Rates*. Ph.D. thesis, Carleton University (2019).

[19] Siamakani SYM, Jiradilok P, Nagai K, Sahamitmongkol R (2020). Discrete mesoscale analysis of adhesive anchors under tensile load taking into account post-installed reinforcement. Constr. Build. Mater..

[20] Neupane CC, Lee J, Pokharel T, Tsang HH, Gad E (2023). Development and challenges in finite element modelling of post-installed anchors in concrete. Struct. Infrastruct. Eng..

[21] Bokor B, Sharma A (2021). Numerical investigations on non-rectangular anchor groups under shear loads applied perpendicular or parallel to an edge. Civ. Eng..

[22] Khalfallah S (2008). Tension stiffening bond modelling of cracked flexural reinforced concrete beams. J. Civ. Eng. Manag..

[23] Gomes H, Awruch A (2001). Some aspects on three-dimensional numerical modelling of reinforced concrete structures using the finite element method. Adv. Eng. Softw..

[24] Khalfallah S (2008). Modeling of bond for pull-out tests. Build. Res. J..

[25] Khaloo, A., Eshghi, I., & Piran, A. P. Study of behavior of reinforced concrete beams with smart rebars using finite element modeling (2010).

[26] Valipour HR, Foster SJ (2010). Finite element modelling of reinforced concrete framed structures including catenary action. Comput. Struct..

[27] Alih S, Khelil A (2012). Behavior of inoxydable steel and their performance as reinforcement bars in concrete beam: Experimental and nonlinear finite element analysis. Constr. Build. Mater..

[28] Matsunaga K, Takase Y, Abe T (2021). Modeling of dowel action for cast-in and post-installed anchors considering bond property. Eng. Struct..

[29] Achillides Z, Pilakoutas K (2006). Fe modelling of bond interaction of FRP bars to concrete. Struct. Concr..

[30] Wu H, Gilbert R (2009). Modeling short-term tension stiffening in reinforced concrete prisms using a continuum-based finite element model. Eng. Struct..

[31] Zanuy C, Curbach M, Lindorf A (2013). Finite element study of bond strength between concrete and reinforcement under uneven confinement condition. Struct. Concr..

[32] Ziari A, Kianoush MR (2014). Finite-element parametric study of bond and splitting stresses in reinforced concrete tie members. J. Struct. Eng..

[33] Tedesco JW, Hughes ML, Ross Ca (1994). Numerical simulation of high strain rate concrete compression tests. Comput. Struct..

[34] Min F, Yao Z, Jiang T (2014). Experimental and numerical study on tensile strength of concrete under different strain rates. Sci. World J..

[35] Yu H, Guo Y, Lai X (2009). Rate-dependent behavior and constitutive model of dp600 steel at strain rate from 10–4 to 103 s-1. Mater. Des..

[36] Lee WS, Lin CF, Liu TJ (2007). Impact and fracture response of sintered 316l stainless steel subjected to high strain rate loading. Mater. Charact..

[37] Spacone E, Limkatanyu S (2000). Responses of reinforced concrete members including bond-slip effects. Struct. J..

[38] Luccioni BM, López DE, Danesi RF (2005). Bond-slip in reinforced concrete elements. Am. Soc. Civ. Eng..

[39] Bao X, Li B (2010). Residual strength of blast damaged reinforced concrete columns. Int. J. Impact Eng..

[40] Shi Y, Li ZX, Hao H (2009). Bond slip modelling and its effect on numerical analysis of blast-induced responses of RC columns. Struct. Eng. Mech..

[41] Shima H, Chou LL, Okamura H (1987). Bond characteristics in post-yield range of deformed bars. Doboku Gakkai Ronbunshu.

[42] Sartipi, S. & Epackachi, S. Finite element modeling of quadruple adhesive bonded anchors under tensile loading. In Tabriz, Iran *11th National Congress on Civil Engineering* (2020).

[43] Ahmed LT, Braimah A (2017). Behaviour of undercut anchors subjected to high strain rate loading. Procedia Eng..

[44] Ahmed LT, Braimah A (2019). Tensile behaviour of adhesive anchors under different strain rates. Eng. Struct..

[45] Ahmed, L.T. & Braimah, A. *Shear Behaviour of Cast-in-Place Anchors at Low and High Strain Rates*. Building Tomorrow’s Society (2018).

[46] Eligehausen, R., Bouska, P., Cervenka, V. & Pukl, R. Size effect of the concrete cone failure load of anchor bolts (1992)

[47] Hallquist, J. O. *et al.* Ls-dyna keyword user’s manual. Livermore Software Technology Corporation. **970**, 299–800 (2007).

[48] Xu M, Wille K (2015). Calibration of k &c concrete model for UHPC in ls-dyna. Adv. Mater. Res..

[49] Weathersby, J. H. Investigation of bond slip between concrete and steel reinforcement under dynamic loading conditions. Louisiana State University and Agricultural & Mechanical College (2003).

[50] CEB-FIP, M. C. Model code for concrete structures. Bulletin D’Information 516 (1990).

[51] Committee, A. Building code requirements for structural concrete (aci 318-08) and commentary. American Concrete Institute (2008).

[52] Ashour A, Alqedra M (2005). Concrete breakout strength of single anchors in tension using neural networks. Adv. Eng. Softw..

[53] Bischoff PH, Perry S (1991). Compressive behaviour of concrete at high strain rates. Mater. Struct..

[54] Fu H, Erki M, Seckin M (1991). Review of effects of loading rate on reinforced concrete. J. Struct. Eng..

[55] Børvik T, Hopperstad O, Berstad T (2003). On the influence of stress triaxiality and strain rate on the behaviour of a structural steel. Part ii. Numerical study. Eur. J. Mech. A Solids.

[56] Lee WS, Lin CF, Liu TJ (2007). Impact and fracture response of sintered 316l stainless steel subjected to high strain rate loading. Mater. Charact..

[57] Odeshi A, Al-Ameeri S, Bassim M (2005). Effect of high strain rate on plastic deformation of a low alloy steel subjected to ballistic impact. J. Mater. Process. Technol..

[58] Malvar, L. J. & Crawford, J. E. *Dynamic increase factors for concrete* (Tech. Rep.; Naval Facilities Engineering Service Center Port hueneme, CA, 1998).

